# Supplementary figures and images for: A DNA metabarcoding approach for recovering plankton communities from archived samples fixed in formalin
Source: PLoS One. 2021 Feb 17;16(2):e0245936. doi: 10.1371/journal.pone.0245936 (PMC7888612; doi:10.1371/journal.pone.0245936)

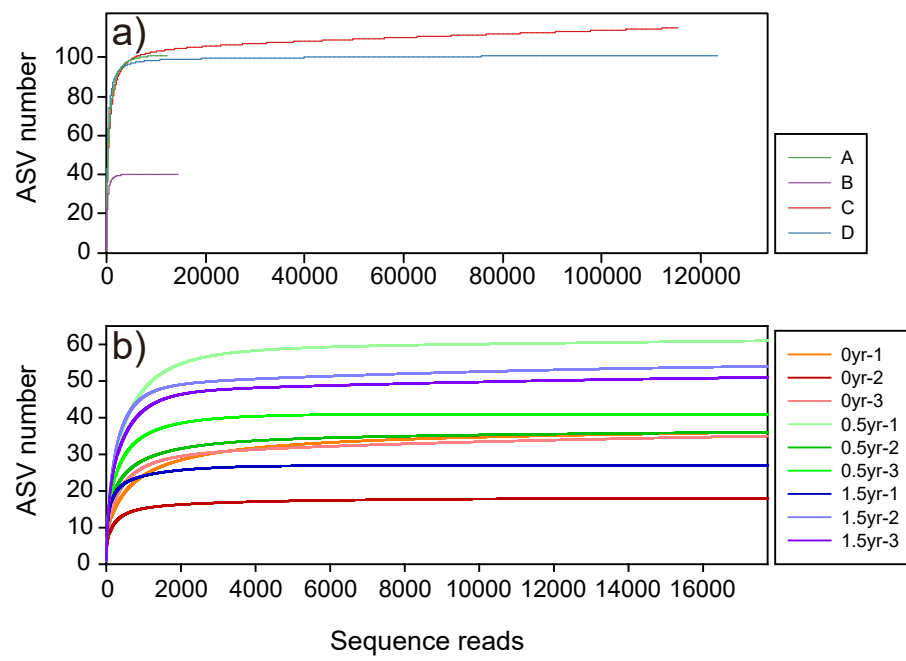

Fig. S1 Rarefaction curves for sample a) A, B, C, and D and b) E for each condition.

Supplement: S1 Fig — (PDF) [file pone.0245936.s001.pdf]
